# Identification of *Salvia haenkei* as gerosuppressant agent by using an integrated senescence-screening assay

**DOI:** 10.18632/aging.101076

**Published:** 2016-12-01

**Authors:** Ivana Matic, Ajinkya Revandkar, Jingjing Chen, Angela Bisio, Stefano Dall'Acqua, Veronica Cocetta, Paola Brun, Giorgio Mancino, Martina Milanese, Maurizio Mattei, Monica Montopoli, Andrea Alimonti

**Affiliations:** ^1^ Laboratory for Research and Development in Aging, Atrahasis S.r.l., 00189 Rome, Italy; ^2^ Department of Chemistry and Pharmaceutical Technologies, University of Genova, 16126 Genova, Italy; ^3^ Research Center, San Pietro “Fatebenefratelli”, 00189 Rome, Italy; ^4^ Animal Technology Facility of University Tor Vergata, 00173 Rome, Italy; ^5^ Department of Pharmaceutical and Pharmacological Sciences, University of Padova, 35121 Padova, Italy; ^6^ Department of Molecular Medicine, University of Padova, 35121 Padova, Italy; ^7^ Studio Associato Gaia Snc, 16121 Genova, Italy; ^8^ Institute of Oncology Research (IOR), Bellinzona CH 6500, Switzerland

**Keywords:** senescence-screening assay, senescence, *Salvia haenkei*, PICS, gerosuppressant

## Abstract

Cellular senescence is a stable cell cycle arrest that is the causative process of aging. The PI3K/AKT/mTOR pathway is implicated in the control of cellular senescence and inhibitors of this pathway have been successfully used for life span prolongation experiments in mammals. PTEN is the major regulator of the PI3K/AKT/mTOR pathway and loss of PTEN promotes a senescence response termed PICS. Here we report a novel-screening assay, for the identification of compounds that block different types of senescence response. By testing a library of more than 3000 natural and chemical compounds in PTEN deficient cells we have found that an extract from *Salvia haenkei* (SH), a native plant of Bolivia is a potent inhibitor of PICS. SH also decreases replicative and UV-mediated senescence in human primary fibroblasts and in a model of *in vitro* reconstructed human epidermis. Mechanistically, SH treatment affects senescence driven by UV by interfering with IL1-ɑ signalling. Pre-clinical and clinical testing of this extract by performing toxicity and irritability evaluation *in vitro* also demonstrate the safety of SH extract for clinical use as anti-aging skin treatment.

## INTRODUCTION

Cells continually experience stress and damage from exogenous and endogenous sources, and their responses range from complete recovery to senescence and cell death [1]. Proliferating cells cannot divide indefinitely due to the progressive shortness of their telomeres and after almost 60 population doublings (Hayflick limit) they stop to grow while remaining metabolically active [1]. Cells can also become senescent prematurely as a result of stressful events such as oncogene over-expression and exposure to DNA damage (for example induced by UV radiation), or oxidative stress (ROS). This phenomenon, referred as premature senescence, occurs rapidly after the triggering event and is a mechanism implicated in cancer and aging. Recent studies have identified a novel type of cellular senescence response that occurs rapidly after inactivation of PTEN, the major regulator of the PI3K/AKT/mTOR pathway in both mouse and human primary cells [2]. Senescence driven by loss of PTEN is mediated by activation of mTOR that actively translate p53, a potent inducer of senescence [2]. Activation of the PI3K/AKT/mTOR pathway independent of PTEN loss is also implicated in replicative senescence, and inhibition of mTOR was shown to prevent ageing in different experimental *in vivo* models [3-6]. Interestingly, rapamycin and metformin two potent mTOR inhibitors, suppress geroconversion, prevent cancer and have minor side effects when administered long-term in anti-aging doses [7-22]. Activation of the PI3K/AKT pathway is also implicated in UV induced cellular senescence, a phenomenon known as photo ageing. Recent findings show that UV irradiation can activate AKT and mTOR, thus boosting senescence and photo aging [23-26]. Considering the need for cost effective active agents that prevent or arrest cellular senescence, efforts have been made to develop an assay for the identification of novel anti-senescence compounds [27, 28]. Natural compounds represent an extraordinary inventory of high diversity structural scaffolds that can offer promising candidates in the major healthcare challenge of delaying ageing [29]. Plant extracts provide a substantial source of potentially active compounds, however so far only few natural compounds have been reported to have anti-senescence effects [30-34]. Based on our previous research results [2], we developed an assay that uses *Pten* null cells as a tool to rapidly identify compounds that decrease senescence in primary cells. Positive hits are later on tested in human primary cells to validate their anti-senescence efficacy in replicative and UV-mediated senescence assays.

Here, we report the results of the screening of more than 3000 substances of both natural (plants and marine extracts) and chemical source. Our data demonstrate that an extract derived from the *Salvia haenkei* (SH) plant is a strong inhibitor of senescence driven by loss of *Pten*, senescence associated to replicative stress and photo aging, both in mouse and human primary cells. Furthermore, we have evaluated *in vitro* the toxicity and irritability of SH on a model of reconstructed human epidermis (EpiSkin) demonstrating SH safety for the human skin and anti-senescence activity.

## RESULTS

### A screening platform for the identification of anti-senescence compounds

**Figure 1:**
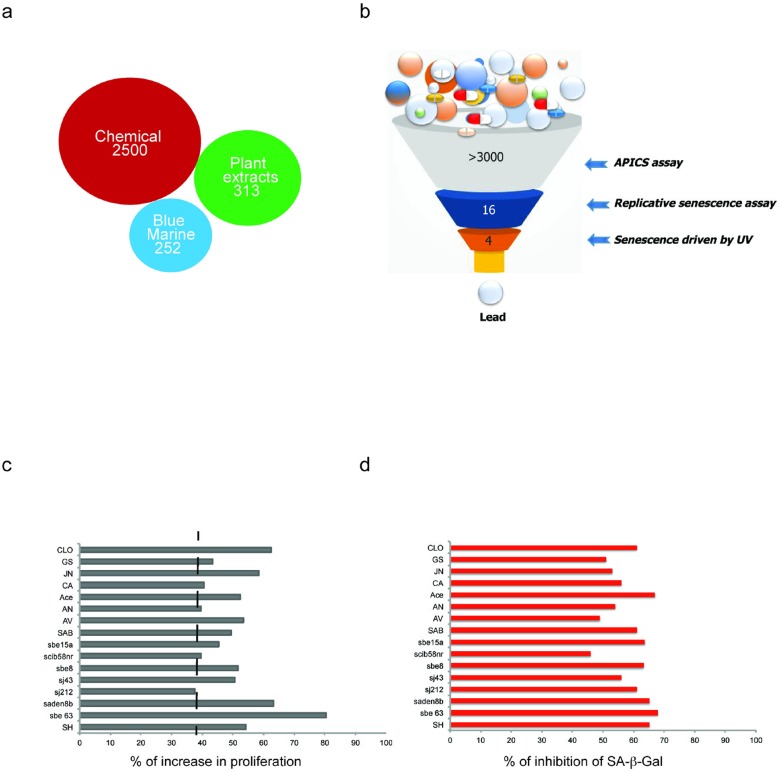
Schematic representation of the platform for the *in vitro* identification of anti-senescent compounds (**a**) Number of chemical and natural extracts from the plants and blue marine ecosystem used for the screening. (**b**) Schematic representation of the screening steps. (**c-d**) Cytostatic and cytotoxic compounds were excluded from the screening and only anti-senescence hits progressed. Compounds that induced a statistically significant increase (of 40% or more) in cell growth were considered potential anti-senescent candidates. Instead, compounds that induced a statistically significant decrease in cell number were considered pro-senescent (40% to 60% decrease), and cytotoxic (more than 60% decrease).

**Figure 2:**
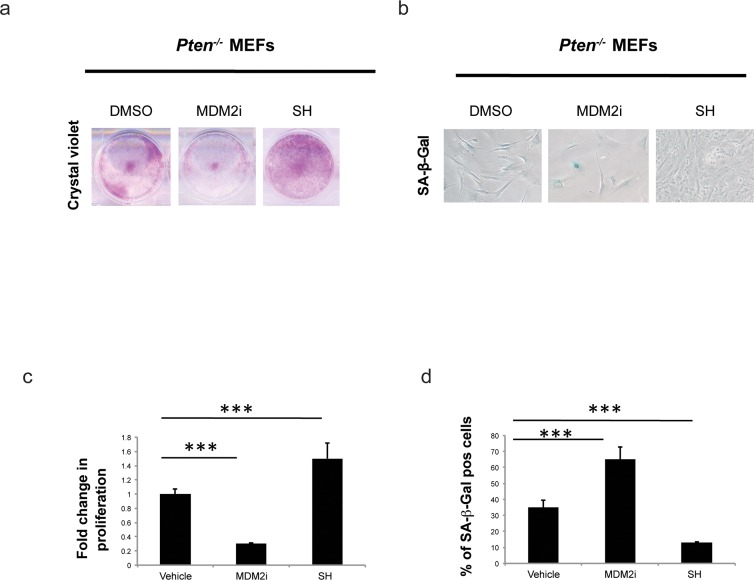
Effect of *S. haenkei* treatment on growth arrest and senescence in Pten−/− MEFs (**a**) Proliferation of Pten−/− MEFs in culture after 5 days of treatment with *S. haenkei* extract. Pten−/− MEFs were plated in concentration of 2×10^4^ cells/ml and treated for 5 days with 10μM MDM2i (Nutlin-3) or 10μg/ml SH extract. After this period, the proliferation was determined using Crystal violet staining. (**c**) Results are expressed as mean values (+SEM) of absorbance at 590nm for duplicates treated with SH and triplicate for control and Nutlin-3 treated groups, from one representative experiment out of 3 independent experiments. (**b-d**) Senescence of Pten−/− MEFs in culture after 5 days of treatment with *S. haenkei* extract. The graph represents percentage of β-galactosidase positive cells revealed in culture upon 5 day treatment with 10μM MDM2i (Nutlin-3) or 10μg/ml *S. haenkei* extract. Quantifications were done on 4 images (roughly 500 cells) per experiment by determining the ratio of perinuclear blue–positive to perinuclear blue–negative cells. Results are expressed as mean values (+SEM) of cell count in three independent experiments.

Loss of *Pten* drives a cellular senescence response in primary cells termed *Pten* loss induced cellular senescence (PICS) [2]. We have recently developed an effective method for identification of pro-senescence compounds to be used for cancer therapy [35]. By modifying this screening assay, we developed a screening platform, for identification of compounds with anti-senescence activity for the treatment of aging and aging-related disorders (Fig. 1). As previously reported [2], upon inactivation of *Pten*, 30-40% of the cells undergo to senescence within 4 days. This provides a screening window to identify hits that affect senescence in a short time frame, something that would be complicated by using a different senescence assay (e.g. replicative senescence). Compounds that decreased the percentage of senescent cells in the screening platform were designated as anti-senescence compounds based on two parameters: 1) cell proliferation and 2) inhibition of SA-β-galactosidase staining (SA-β-gal), a prototypical senescence marker [36]. For the identification of new anti-senescence hits, the library was created from 3065 substances comprising 1) chemical molecules (2500) 2) blue marine extracts (252) 3) plant extracts (313) as reported in Fig. 1a. *Pten* lx/lx MEFs were infected with a retro-viral Cre vector to delete *Pten* and selected for two days with puromycin to obtain *Pten−/−* MEFs (t0). Experimentally, the screening was carried out in three steps using *Pten−/−* MEFs (Fig. 1b and Supplementary Fig. S1a). In the first step, compounds were studied in triplicates using a single concentration (10 μg/ml). Cells were treated for 5 days from t0. Candidate compounds that increased the cell growth rate of more than 30% compared to control (n=80/3065), were considered as potentially anti-senescent hits and were retested in triplicate. Validated hits (n=54/80) were tested for SA-β-galactivity subsequently. Compounds that decreased the SA-β-gal staining more than 30% (compared to DMSO treated *Pten*^−/−^ cells), passed this filter. Among these hits there were 11 extracts from plants, sponges and marine bacteria and 5 chemical compounds, demonstrating that nature is a valuable source of biologically active phytochemicals that can slow down senescence (Fig. 1c, d). Interestingly, 1/16 hit was plant extract of Angelica whereas, 8/16 hits were plant extracts of Salvias, the largest genus of plants in the family Lamiaceae, with the number of species estimated to range from 700 to nearly 1,000 members. 2/16 hits were from a sponge extract and a marine bacterium associated to a sponge respectively, whereas 5/16 hits were small molecule inhibitors (Supplementary Table S1a). The most potent of these hits was an extract of *Salvia haenkei* (SH) a member of the family of Salvias, native of Bolivia. SH decreased senescence of 50% when compared to untreated control, by-passing the growth arrest promoted by *Pten* loss (Fig. 2a-d). Interestingly, *Pten* null cells treated with SH had a growth rate similar to *Pten* wt cells. *Pten* wt cells also, did not significantly increase proliferation after SH treatment when compared to vehicle treated control (Supplementary Fig. S1b). HPLC analysis revealed that SH extract contained high levels of apigenin and luteolin glycosides, two flavonoids with anti-cancer properties [37] (Supplementary Fig. S1c and Supplementary Table S1b).

### Identification of compounds that prevent replicative and radiation-driven senescence

**Figure 3:**
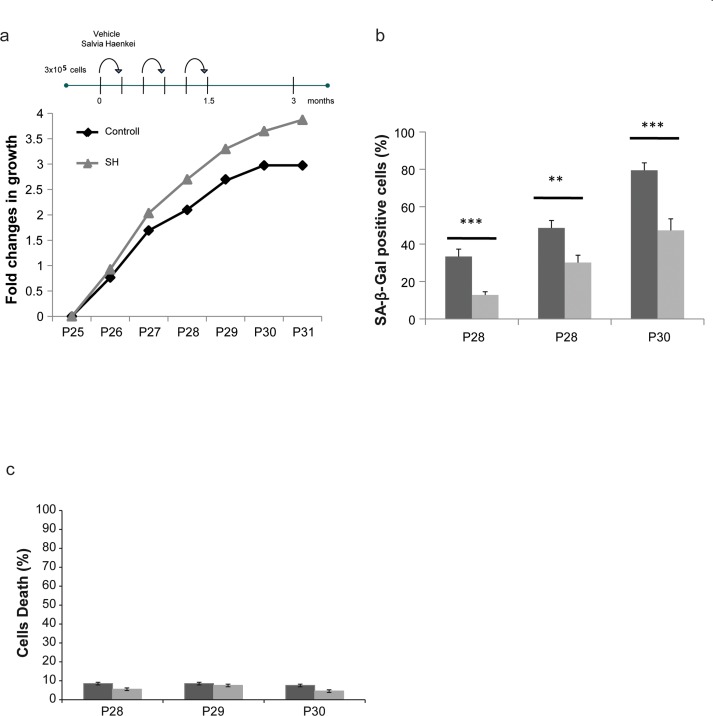
Effect of *S. haenkei* treatment on replicative senescence in human fibroblasts (**a**) Growth curve of human WI38 fibroblasts treated with *S*. *haenkei* extract. WI-CCL75 human fibroblasts were plated 300.000 cells per 10cm dish, and subsequently passed and replated in the same number every 3 days for total of 24 passages up to the point when treatment with *S. haenkei* was initiated. At passage 25, cells were plated at the same number 300.000 cells per plate, and treated with 10μg/ml SH extract. Every 3 days cell number was determined by Trypan blue staining and cells replated 300.000 per plate and re-treated. Results are expressed as fold change in cell number from one representative experiment out of 4 independent experiments. (**b**) Senescence of human WI38 fibroblasts treated with *S. haenkei* extract. The graph represents percentage of β-galactosidase positive cells revealed in culture at each passage. Quantifications were done on 4 images (roughly 500 cells) per experiment by determining the ratio of perinuclear blue–positive to perinuclear blue–negative cells. Results are expressed as mean values (+SEM) of cell count in four independent experiments. (**c**) Cell death in culture of human WI38 fibroblasts upon treatment with *S. haenkei* extract. The graph represents percentage of Trypan blue positive (dead) cells revealed in culture at each passage. Quantifications were done on one experimental image (roughly 100 cells) in one representative experiment.

**Figure 4:**
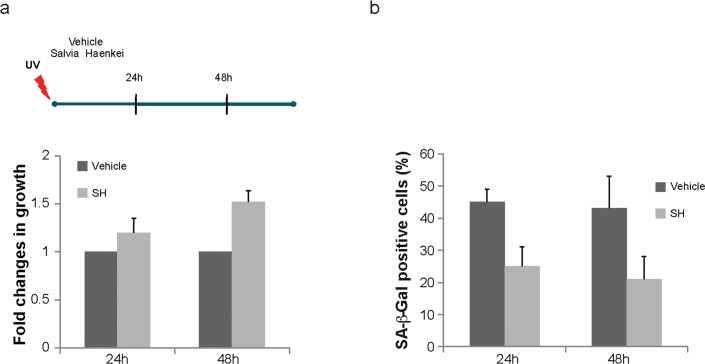
Effect of S. *haenkei* treatment on photo ageing of human fibroblasts WICCL75 human fibroblasts were irradiated with 30J/m^2^ UVB and 3h later treated with 10μg/ml *S. haenkei* extract. (**a**) Proliferation of irradiated human WI38 fibroblasts treated with *S. haenkei* extract. Cell proliferation was measured by Crystal violet staining at time points treatment (10μg/ml) 24h and 48h and represented as fold change in growth (compared to untreated control). Results are expressed as mean values (+SEM) for duplicate in each group in one representative experiment out of three independent experiments. (**b**) Senescence of irradiated human WI38 fibroblasts treated with *S. haenkei* extract. The graph represents percentage of β-galactosidase positive cells revealed in culture at time points 24h and 48h. Quantifications were done on 4 images (roughly 500 cells) per experiment by determining the ratio of perinuclear blue–positive to perinuclear blue–negative cells. Results are expressed as mean values (+SEM) of cell count in three independent experiments.

To assess whether identified hits decrease replicative senescence *in vitro*, we used human dermal fibroblasts. To this extent we carried on a series of experiments using the 3T3 protocol in the WI38-CCL75 cells for a period of over 3 months. Cells were plated and subsequently passed and re-plated in the same number every 3 days, in the presence or absence of selected hits. Only four out of 16 hits (2 plants and 2 marine extracts) were able to decrease replicative senescence and were further developed in our screening cascade. Among these extracts, SH showed again the most relevant activity (Supplementary Fig. S2a). As represented in Fig. 3a, while untreated cells stopped growing at passage 30, cells treated with SH continued to proliferate. Moreover, senescence in treated cells was significantly decreased when compared to control as assessed by the SA-β-Gal staining (Fig. 3b). The reduction in the percentage of SA-β-Gal staining in these cells was comparable to the one observed in *Pten* null MEFs showing a correspondence between these two models. Importantly, treatment of cells with SH for a period of three months was not associated to increased cell death, as demonstrated by the cell viability assay (Fig. 3c). Taken together, these data demonstrate that SH extract is a potent suppressor of PICS and replicative senescence in human primary dermal fibroblasts. Next, we assessed the anti-senescence activity of SH in an assay of senescence driven by UV irradiation. We set up experimental conditions to induce premature senescence using UV irradiation in WI38-CCL75 human fibroblasts and assess senescence by performing SA-β-Gal staining at 24 and 48 hours after irradiation. SH treatment was able to prevent growth arrest and senescence in irradiated fibroblasts already at 24h after treatment (Fig. 4a, b). At a later time point (48h) the effect of SH resulted even more efficient, mildly stimulating the proliferation of the control cells as well. Taken together, these data demonstrate that SH is a powerful anti-senescence agent in PICS, replicative senescence and cells treated with UV irradiation.

### *Salvia haenkei* extract reduce oxidative stress mediated by H_2_O_2_

**Figure 5:**
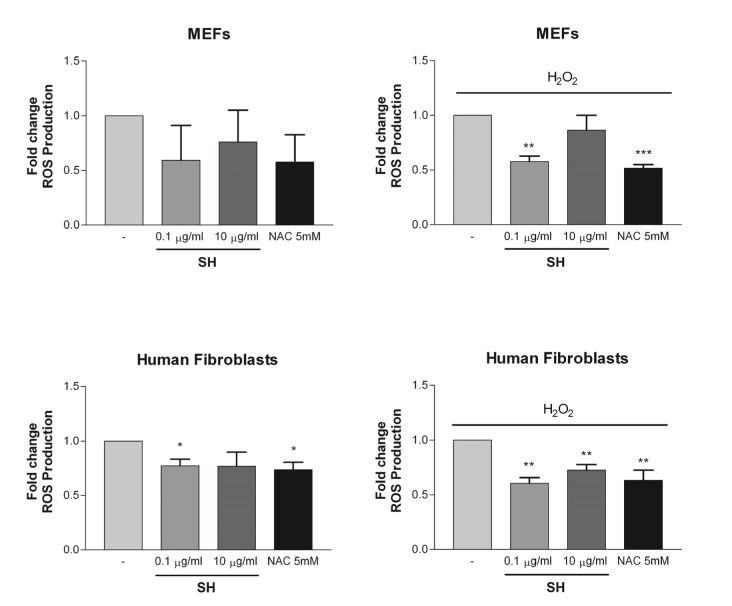
Effect of S. *haenkei* treatment on ROS production on MEFs and human fibroblasts ROS generation were measured after 3 hours of incubation in untreated cells and after H_2_O_2_ exposure. Treatment with NAC was used as positive control. Data are expressed as mean ± SEM percentage of basal (100%) DCF fluorescence intensity (FI) of three independent experiments. **p<0.05 treated *vs* untreated. (**a**) ROS production in unstressed MEFs; (**b**) ROS production in MEFs after exposure to H_2_O_2;_ (**c**) ROS production in unstressed human fibroblasts; (**d**) ROS production in human fibroblasts after exposure to H_2_O_2_.

Next, we assessed the efficacy of SH in cells undergoing to oxidative stress. While ROS are produced as a product of normal cellular functioning, excessive amounts can cause deleterious effects. Oxidative stress also promotes cellular senescence and premature aging in the skin [38]. MEFs and human dermal fibroblasts were treated with H_2_O_2_ - a potent inducer of ROS. The antioxidant activity of SH (0.1-10μg/ml) was assayed in MEFs and human fibroblasts immediately after the exposure to H_2_O_2_. Interestingly, SH treatment in MEFs reduced the intracellular levels of ROS both in untreated cells and in cells treated with H_2_O_2_. The effect of SH in these cells was similar to that of N-acetylcysteine (NAC), a known antioxidant compound clinically used to prevent the accumulation of ROS in different inflammatory conditions [39] (Fig. 5a, b). A similar effect was also observed in human fibroblasts treated with and without H_2_O_2_ (Fig. 5c, d). Taken together, these data demonstrate that SH treatment decreases the intracellular levels of ROS thereby explaining its efficacy in preventing different types of senescence.

### *Salvia haenkei* treatment decreases senescence in a human 3-D skin model (EpiSkin), by interfering with IL1α secretion

**Figure 6:**
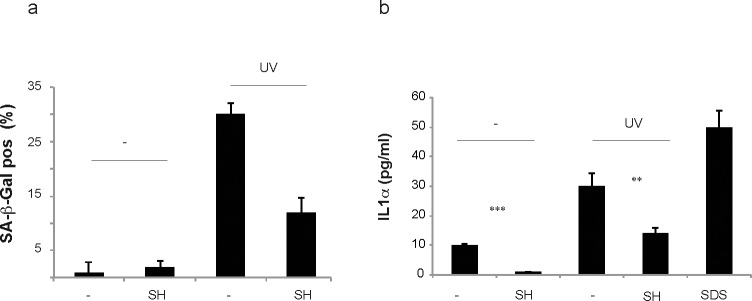
Toxicity and irritability evaluation of *S. haenkei* extract in reconstituted human epidermis Skin issues were cultured in 12 well plates containing 37°C pre-warmed maintenance media (2 ml/well) and incubated overnight at 37°C, 5% CO_2_ and 95% humidity, prior to the experiment. EpiSkin tissues were irradiated with UVB (30J/m^2^) and 3 hours later treated by topical application with 10μg/ml *Salvia haenkei* extract and 5% SDS for positive control. 4h later, the epidermis was washed with PBS and left for incubation at 37°C, 5% CO_2_. (**a**) 22h after topical application of 10μg/ml of SH on EpiSkin tissues, senescence (bars) was calculated as a percentage of the control for β-galactosidase positive cells. Here, the treatment with UV was used as positive control. Results are expressed as the mean (+SEM) of triplicates in one representative experiment. (**b**) IL1ɑ production by EpiSkin tissue in response to *S. haenkei* treatment in the presence or absence of UV irradiation. Treatment with SDS was used as positive control. 22h after topical application of 10μg/ml of S*. haenkei* extract on EpiSkin tissues, supernatants were collected and samples stored at −80°C. The levels of IL1α were tested by ELISA. Results are represented in logarithmic values (pg/ml) and expressed as mean value±SEM, from triplicates in one experiment.

To assess the efficacy and safety of SH in a human skin model we took advantage of the EpiSkin model that has been recognized as a valid alternative to animal test procedures [40]. EpiSkin is an *in vitro* reconstructed human epidermis from normal human keratinocytes cultured on a collagen matrix at the air-liquid interface. This model is histologically similar to the *in vivo* human epidermis [41]. To assess the anti-senescence potential and toxicity of SH extract following topical treatment we delivered SH in an oil-in-water conventional skin care vehicle. Human epidermis was treated with UV in the presence or absence of SH (10μg/ml) and SA-β-Gal staining was assessed 42h after treatment. Quantification of SA-β-Gal staining showed that SH decreased the number of senescent cells in the human epidermis validating our previous results (Fig. 6a). Next, we checked the levels of IL-ɑ secreted by the human epidermis in the culture media of samples treated with UV +/− SH. Recent findings demonstrate that IL1ɑ is an essential regulator of paracrine senescence since it can control the senescence-associated secretory phenotype (SASP) [42]. Indeed, senescent cells can release IL1ɑ in the microenvironment to promote senescence in normal cells. This phenomenon has been proposed as the cause of the progressive increase of senescent cells in normal tissues during aging. Surprisingly, SH treatment suppressed also the levels of IL1ɑ released by the human epidermis after treatment with UV and this correlated with a decreased SA-β-Gal staining (Fig. 6a and b). Taken together, these data demonstrate that SH treatment decreases paracrine senescence by interfering with IL1&alpha; released by senescent cells. A cell viability assay excluded any cytotoxic activity of SH in cells treated with this compound also in this model (data not shown). Note that as control for this experiment, we used SDS treatment. SDS is an irritant known to promote the secretion of IL1ɑ. Therefore, these data also demonstrate that SH treatment does not irritate human skin at the concentration of 10μg/ml.

## DISCUSSION

Cellular senescence is a stable cell growth arrest that occurs in almost all the cells of human tissues during aging [1]. By definition, senescent cells remain arrested even in the presence of growth factors, but are metabolically active and stain positive for SA-β-gal at pH6, a marker of enhanced lysosomal activity [36]. Senescent cells can also release in the tissue microenvironment several factors known collectively as the senescence associated secretory phenotype (SASP) [1]. These factors can also propagate senescence to neighboring cells, a process known as paracrine senescence. Through the SASP, senescent cells can induce deleterious effects on normal tissues. Mice, whose senescent cells were killed off, were healthier than transgenic mice in which these cells accumulated as effect of aging. Kidneys and heart function in these mice were enhanced; moreover, they were less prone to develop cancers than control animals [43]. Thus, therapies that prevent the accumulation of senescence cells in normal tissues or that selectively kill senescent cells (senolytic therapies) could be used for the treatment of aging and aging associated disorders such as cancer, neurodegenerative and cardiovascular diseases. Eliminating senescent cells could also extend the lifespans of health subjects as recently demonstrated in the mice [43]. We have previously identified a novel type of senescence response, which occurs rapidly after inactivation of PTEN, an essential regulator of the PI3K/AKT/mTOR pathway in both mouse and human cells [2, 44]. Several evidence demonstrates that the PI3K/AKT pathway is implicated in different types of cellular senescence response, including replicative senescence, oncogene-induced senescence (OIS) and photoaging [2, 44, 45]. Inhibition of mTOR, a crucial downstream component of this pathway, attenuates senescence and prolongs the life span of mice [7, 46]. This effect is probably due to the attenuation of the SASP rather than to telomeres preservation as demonstrated by recent findings. Indeed, mTOR inhibition blocks some of the negative effects of the SASP and these compounds may therefore be clinically developed for the prevention of aging or aging related disorders [47, 48]. Currently, there is a high demand for compounds of natural origin that can block senescence to be used as gerosuppressants. Using *Pten* null cells as a model, we have developed a screening assay to identify compounds that block senescence. Inactivation of the tumor suppressor PTEN promotes a strong cellular senescence response that limits the replicative lifespan of primary cells, which exhibit a characteristic enlarged and flattened morphology and increased SA-β-Gal activity. Without ideal means to develop a completely automated screen, we developed a pragmatic semi-automated approach using a high-throughput screening followed by visual assessment of cells of interest, achieved by crystal violet and SA-β-Gal, two standard and accepted assays used to identify senescent cells [49]. The use of these 2 assays in Pten null cells greatly reduces the time and resources required to make the initial identification of potential hits, and although not completely automated, these methods are nonetheless rapid. Herein, we confirm that in contrast to chemical compounds, which showed very little percentage of active anti-senescence compounds, natural compounds affected cellular senescence with much higher rate. The identification of natural compounds as regulators of anti-senescence and anti-oxidant is critical in the discovery of novel therapeutics. Using this screening platform, we have identified *Salvia haenkei* as anti-senescent plant extract and tested it for replicative senescence and photo ageing prevention. Importantly, our experiments in a model of skin human epidermis (EpiSkin) demonstrate that SH extract can decrease the levels of senescence cells by affecting the secretion of IL1ɑ. IL1ɑ is considered the master regulator of the SASP and a recent paper demonstrates that compounds that interfere with IL1 signaling blocks replicative senescence, OIS and PICS [49,50]. Taken together, these data suggest that SH extract may be safely used in a skin-care preparation to prevent skin aging also in consideration of the found SH anti-microbial activity (Supplementary Fig. S2b). Replicative and UV-mediated senescence in skin are responsible of wrinkling, pigment changes, cracking and loss of elasticity among others. Acute dermal overexposure to UV radiation also causes an inflammatory response, erythema and leukocyte infiltration [51]. Oxidative stress initiated by ROS generation is also an important mediator of cellular aging, including skin aging. As demonstrated here, SH treatment also decreased ROS levels in cells treated with H_2_O_2_ at early and late time points. Finally, experiments in the EpiSkin model also demonstrate that a skin care preparation containing SH extract is safe and not irritant for the human skin. In sum, our findings describe novel screening assays for the identification of gerosuppressant agents. Previous screenings have reported the identification of anti-aging compounds using different biological systems (e.g. yeast) and assays (e.g. in vitro assays, computer screening). Since the pathways that control aging in mammals have homologs in yeast, flies, and worms, several of these screenings have been performed in invertebrates instead that in mammalian cells [52-54]. These screenings have contributed to the identification of several gerosuppressants active compounds such as rapamycin, metformin and resveratrol whose efficacy have been later on validated in mammalian cells. Our screening based on the use of PTEN deficient mouse embryonic fibroblasts in a first step and in the consecutive validation of positive HITs in human cells offers a promising alternative to these models for the rapid identification of effective gerosuppressants.

## MATERIALS AND METHODS

### Plant material and preparation of plant extract

The extract of *Salvia haenkei* was kindly provided by Dr. Bisio from Dept. of Chemistry and Pharmaceutical Technologies, University of Genoa, Italy. The plant material was harvested and leaves were put in a ventilated stove at 45°C for 24 hours, and then ground as fine powder using a mixer IKA universal M20. A quantity of 20.0g of powdered dried plant was weighed in a 100ml conical flask to which 70ml of hexane (purity 99%) was added for the pre-extraction. The flask was placed in a bath sonicator (Branson 8210) and sonicated at a temperature of 40°C for 30 minutes. The mixture was filtered with filter paper, followed by washing with 20ml of hexane and then with 50ml of hexane. The filtrate was poured into a flask and the solvent was concentrated under vacuum (about 11mmHg) up to 5-10 ml by rotavapor, using a water bath at 40°C. This residue as poured into a glass container followed by evaporation of the solvent. The filtrate was left open overnight in a well-ventilated hood until complete evaporation of the last traces of solvent. The solids collected on the filter, were divided and air-dried overnight in the hood. The dried material is extracted in the same way with methanol-water (90:10). The dried material from the filters was dissolved in 70ml of 90% methanol. The mixture was sonicated at 40°C for 30 minutes, after being filtered, then washed with 20 ml of 90% methanol. The filtrate was poured into a flask and the solvent completely evaporated under vacuum. The dry extract was dissolved in 90% methanol in the least possible amount of absolute methanol, using sonication and poured into a glass container to evaporate overnight in the hood. The extract was reconstituted with pure DMSO at a concentration of 10mg/ml and kept at −20°C until dilution for the treatment of cell cultures. The SH extract was analysed by HPLC-DAD and HPLC-MS obtaining a phytochemical fingerprint. The identified constituents are summarized in supplementary data (Supplementary Fig. S1c and Supplementary Table S1b).

### MEFs isolation

Pten^lx/lx^ MEFs were prepared as previously described [36]. Briefly, pregnant female mice at day 13 postcoitum (assuming as day one the first day the plug was observed) were sacrificed by cervical dislocation. The uterine horns were dissected out, briefly rinsed in 70% (v/v) ethanol and placed into a petri dish containing PBS (Gibco 14190-169, without bivalent cations). Each embryo was separated from its placenta and surrounding membranes, the brain and dark red organs were cut out. Embryos were washed with fresh PBS, removing as much blood as possible. Using a minimal amount of PBS and razor blades, the embryos were finely minced into a suspension of cells to which several ml of trypsin-EDTA (about 1-2ml per embryo, Gibco 25300-096) was added. Following incubation with gentle shaking at 37°C for 15min the resulting cell suspension was pelleted and resuspended in fresh DMEM (ReadyMix, PAA) containing 10% FCS, 2mM L-glutamine, 2mM penicillin, 50μg/ml streptomycin. Cells were plated out at 1 embryo equivalent per 10cm dish (“passage No. 0”). The adherent fibroblasts reached confluence at day 4 when they were collected and stored at −80°C prior to use in the APICS assay (for details of this assay see also Fig. 1 and Supplementary Fig. S1a)

### Cell cultures and infections

Pten^lx/lx^ MEFs were isolated as described previously [36]. To produce Pten^−/−^ MEFs, Pten^lx/lx^ MEFs were subsequently infected with a viral vector retro- Cre - recombinase (Adgene Plasmid pMSCV PIG Cre (Cre IRES Puro vector)). This retro-Cre was produced by transfection of Phoenix cells (Eco and Ampho from Life Technologies) at 70-80% confluence using Lipofectamine 2000 (Invitrogen). At 70% confluence, Pten^lx/lx^ MEFs were infected with supernatant from Phoenix cells, collected after 48h of transfection with retro-Cre vector. To increase the efficiency of infection 5μg/ml Polybrene (Santa Cruz) was used. 12h after the first infection, Pten^lx/lx^ MEFs infection was repeated. 24h later, infected Pten^lx/lx^ MEFs were selected with 3μg/ml puromycin. 48h later, Pten^−/−^ were plated and treated with compounds within APICS molecular screening assay. As control (Pten^wt^) cells in APICS assay we used Pten^lx/lx^MEFs infected with a viral vector retro- PIG (Adgene plasmid pMSCVPIG (Pure IRESGFPvector)), resistant to puromycin, by following the same protocol as described for Pten^−/−^ MEFs. Human WI38-CCL75 fibroblast cell line (ATCC) was used for 3T3 assay and UV irradiation experiments. All cell cultures were maintained in fresh DMEM (ReadyMix, PAA) containing 10% FCS, 2mM L-glutamine, 2mM penicillin, 50μg/ml streptomycin.

### Cell proliferation and viability

Cell proliferation was measured using staining with Crystal violet colour (Sigma Aldrich). Cells were per-fixed with 4% formaldehyde for 15min., washed with PBS and stained with 0.1% Crystal violet for 20 minutes. After 3 wash cycles with PBS, cells were lysed in 10% acetic acid and color intensity read at 590nm on SUNRISE ELISA reader (Tecan, Switzerland). Growth curve analysis was carried out as previously described in literature [55]. Cell viability was assessed using Trypan blue exclusion.

### SA-β-galactosidase assay

Senescence staining was performed using the commercial Senescence Detection Kit (Calbiochem, #JA7633), designed to histochemically detect β-gal activity in cultured cells at pH 6.0. β-gal at pH 6.0 is present only in senescent cells and is not found in presenescent, quiescent, or immortal cells. Standard protocols were followed [56], and quantifications were done on 4 images (roughly 500 cells) per experiment by determining the ratio of perinuclear blue–positive to perinuclear blue–negative cells. Fluorescent nuclear staining was performed using 4′,6-diamidino-2-phenylindole (DAPI), purchased from Sigma Aldrich.

### 3T3 protocol

Human primary fibroblasts WI38-CCL75 were plated in 10cm^2^ dishes (3×10^5^cells/dish), and subsequently passed and re-plated in the same number every 3 days for total of 24 passages up to the point when treatment was initiated. At passage 25, cells were plated at the same number and treated with the SH extract in single concentration (10μg/ml). Every 3 days cell number was determined by Trypan blue counting, cells re-plated and re-treated. At passages 28, 29 and 30 senescence was evaluated by measuring β-gal expression.

### UV irradiation assay

We tested SH extract for the ability to prevent senescence in a model of UVB irradiated human fibroblast primary cells. To this purpose, WI38-CCL75 human fibroblasts were irradiated with the optimized non cytotoxic dose (30J/m^2^) of UVB irradiation that causes senescence. 3h after irradiation, positive hits were added in single concentration (10μg/ml). Cell proliferation was determined at different time points using crystal violet assay. Senescence was measured by β-gal expression.

### ROS production

ROS were quantified using 2′,7′-dichlorofluorescin-diacetate (H_2_-DCF-DA, Sigma-Aldrich), as previously described [57]. Upon cleavage of the acetate groups by intracellular esterase and oxidation, the H_2_-DCF-DA is converted to the fluorescent 2′,7′-dichlorofluorescein (DCF). Briefly, the cells (5×10^3^) were seeded into 96-well plates and allowed to adhere overnight. ROS level was measured after the exposure to SH extract for 3 hours in the absence or presence of H_2_O_2_, and subsequent addition of 50 μM H_2_-DCF-DA, further incubation for 30 min at 37°C and washing with phosphate-buffered saline (PBS). DCF fluorescence intensity was measured at excitation 485 nm—emission 535 nm, using a Multilabel Plate Reader VICTOR X3 (PerkinElmer). Fold increase in ROS production was calculated using the equation: (F_treatment_—F_blank_)/(F_control_—F_blank_), where F is the fluorescence reading.

### EpiSkinLM

The EpiSkinLM model (LM: large model; manufactured by EPISKIN S.N.C., Lyon, France) is a reconstructed organotypic culture of human adult keratinocytes that reproduce a multilayered and differentiated human epidermis. Briefly, human adult keratinocytes were seeded on a dermal substitute consisting of a collagen I matrix coated with a layer of collagen IV fixed to the bottom of a plastic chamber. Epithelial differentiation was obtained by an air-exposed step leading to a 3-dimensional epidermis construct (1.07cm^2^ surface), with basal, spinous, granular layers (with specific markers) and a stratum corneum. EpiSkinLM units were delivered to the laboratory within 24 hours after preparation. Upon arrival, tissues were transferred to 12 well plates containing 37°C pre-warmed maintenance media (2 ml/well) and incubated overnight at 37°C, 5% CO_2_ and 95% humidity. Skin units were treated with 30J/m^2^ of UVB irradiation. SH extract (10μg/ml) was formulated in a standard oil emulsion and applied topically to the surface of the epidermis. 4h after UV irradiation, the epidermis was washed with PBS and left for incubation at 37°C, 5% CO_2_. 42h later, supernatants were collected and stored at −80°C. To assess toxicity of the extract, cell viability test was performed using MTT assay (In Vitro Toxicology Assay Kit, Sigma Aldrich) (data not shown) and to assess the release of IL-1α, we analysed collected supernatants by ELISA (Abcam) for presence of IL-1α. For the quantification of senescence in the EpiSkinLM modelfrozen sections of skin units (6μm thick) were stained for SA-β-Gal as described above, 42h after irradiation +/− SH treatment at (10μg/ml).

### Cytokine assay

Supernatants of EpiSkin epidermis, derived in different conditions (negative control-PBS, positive control-SDS and treatment with SH extract 10μg/ml) were collected and stored at −80°C. IL-1α (limit of sensitivity < 10 pg/ml) levels were determined by ELISA kit (Abcam) according to the manufacturer's specifications. Results are expressed as pg/ml and reported as means from three independent experiments.

### Statistical analysis

All values obtained are means of at least three independent experiments performed in duplicate or triplicate. Results are presented as mean value ± SEM. Control and treated groups were compared using the analysis of variance (ANOVA) test. In all analyses, a p-value of <0.05 was considered statistically significant. Data were processed using Assistat (version 7.6b) and Microsoft Excel software.

## SUPPLEMENTARY MATERIALS FIGURES AND TABLE


