# Supplementary material for: Identification of Salvia haenkei as gerosuppressant agent by using an integrated senescence-screening assay
Source: Aging (Albany NY). 2016 Dec 1;8(12):3223–36. doi: 10.18632/aging.101076 (PMC5270665; doi:10.18632/aging.101076)
Supplement: Supplementary file 1 [file aging-08-3223-s001.pdf]

SUPPLEMENTARY MATERIALS

Supplementary Methods

HPLC-MS and HPLC-DAD-ELSD quali-quantitative analysis

Qualitative and quantitative constituents of *Salvia haenkeii* were performed by high-performance liquid chromatography-tandem mass spectrometry (HPLC-MS) and high-performance liquid chromatography coupled with a diode array detector and an evaporative light scattering detector (HPLC-DAD-ELSD). For HPLC-MS analysis a Varian 212 binary chromatograph equipped with 500MS ion trap and Prostar 430 autosampler was used (Varian Inc., USA). For the HPLC-DAD-ELSD analysis an Agilent 1100 Series chromatograph with 1100 Diode Array detector and Sedex LX 60 Evaporative Light Scattering Detector (ELSD) was used. As stationary phase an Agilent Eclipse XDB-C8 2.1 x 150 mm, 3.5 µm (Agilent Tecnologies, USA) was used. Quantification of phenolic constituents was obtained with the method of calibration curve: rutin (Sigma Aldrich, St. Louis, MO, USA) was used as external standard for flavonoid quantification. Calibration curves were Y = 144232X + 112 (R2 = 0.9998) for rutin.

Microbiological assessments

Streptococcus pyogenes and Staphylococcus aureus were isolated from clinical specimens of human skin, cultured on agar plates and identified according to the appearance of colonies, growth conditions and metabolic enzymatic activities. Bacteria were grown in lysogeny broth (LB) at 37°C until mid-log phase determined by spectrophotometric analysis, reading the optical density (O.D.) at 600 nm. Bacterial cultures were then diluted in LB to reach concentration of $1x10^6$ colony-forming units (CFU)/mL and incubated at 37°C for 16 hours with SH at the indicated concentrations. At the end of incubation, bacterial load was estimated by reading the O.D. using a spectrophotometer (Sunrise, Tecan; Switzerland). Data were expressed as mean ± standard error of the percentage of O.D. calculated versus the respective untreated samples.

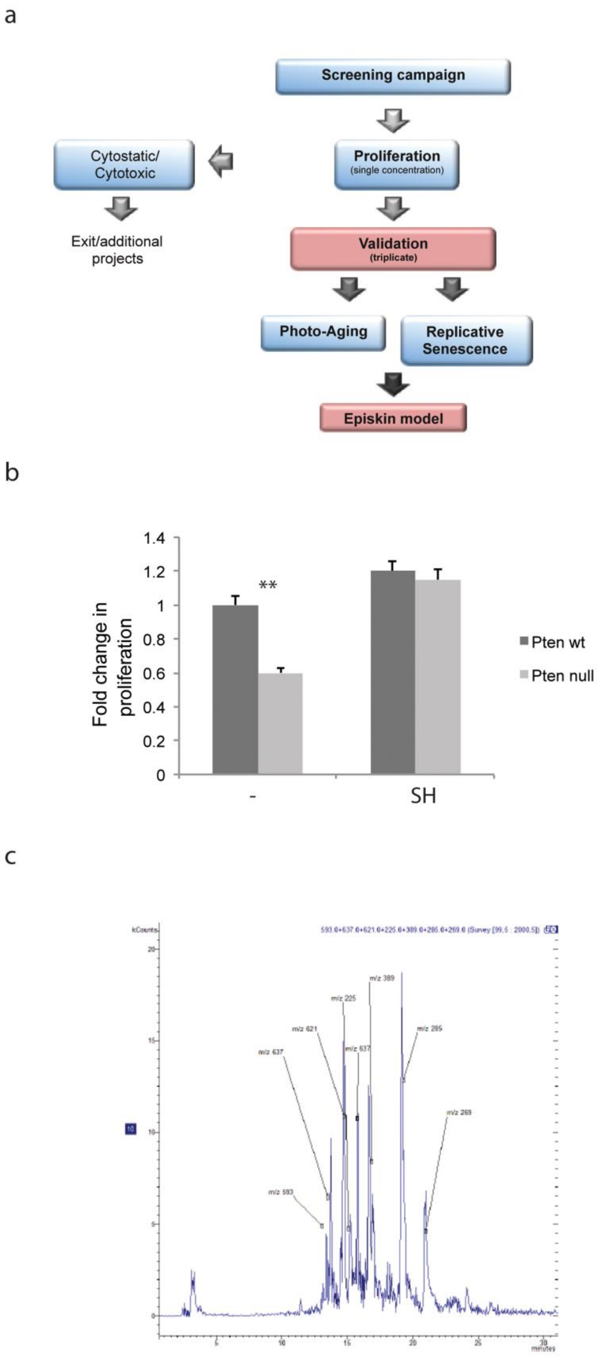

Supplementary Figure S1. (**a**) Schematic representation of PICS assay First step is the identification of candidate compounds that have an anti-senescent effect in the in vitro PICS assay (screening campaign). Compounds are tested for their effect on proliferation and b-gal expression in primary murine embryonal fibroblasts (MEFs) lacking *Pten* gene (*Pten* null). The candidate compounds that are able to increase growth rate more than 40% compared to control and at the same time block senescence (determined by less b-gal positive cells), are selected for testing their ability to block photo-induced and replicative senescence in human fibroblasts (validation). Cytotoxic compounds instead, are selected for external projects on anti-tumor therapy. Candidate compounds that demonstrate efficiency in prevention of replicative and photo ageing are tested in reconstituted skin model as part of preclinical development. Successful (non toxic and non irritant) candidates are formulated into topical products and proceed to clinical trials. (**b**) Proliferation of *Pten* null MEFs in culture after 5 days of treatment with *S. haenkei* extract. *Pten* null MEFs were plated in concentration of $2x10^4$ cells/ml and treated for 5 days with 10µM Nutlin-3 or 10µg/ml SH extract. After this period, the proliferation was determined using Crystal violet staining. (**c**) An exemplificative chromatogram is reported with the indication of twelve compounds identified on the basis of MSn fragmentations.

a

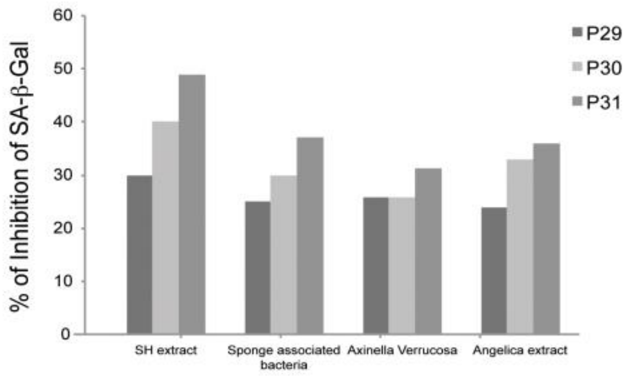

b

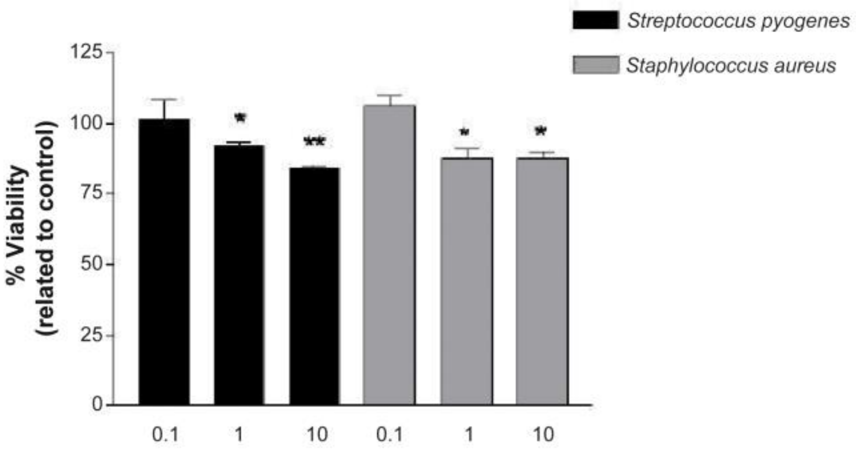

Supplementary Figure S2. (**a**) To assess replicative senescence *in vitro*, 3T3 protocol was performed on human dermal fibroblasts (WI38-CCL75 cells, ATCC). Cells were plated and subsequently passed and re-plated (step called passage) in the same number every 3 days, for a period of over 3 months. From passage 25, 3T3 protocol was continued, but in the presence or absence of selected hits (represented here SH extract, sponge associated bacteria, *Axinella Verrucosa* and *Angelica* extract). The graph represents percentage of β-galactosidase positive cells revealed in culture on passages 29, 30 and 31. Results are expressed as mean values (+SEM) of cell count in three independent experiments. (**b**) Antimicrobic effect of *Salvia haenkeii* treatment on Streptococcus pyogenes and Staphylococcus aureus. Antimicrobic activity was measured after 16 hours of exposure. At the end of incubation, bacterial load was estimated by reading the O.D. using a spectrophotometer. Data were expressed as mean ± standard error of the percentage of O.D. calculated versus the respective untreated samples. *p<0.05 treated vs untreated.

a

Code	Hit description
SH	crude extract of Salvia haenkei
sbe 63	pure extract of Salvia blepharophylla
saden8b	pure extract of Salvia adenophora
sj212	pure extract of Salvia jamensis
sj43	pure extract of Salvia jamensis
sbe8	pure extract of Salvia blepharophylla
scib58nr	pure extract of Salvia cinnabarina
sbe15a	pure extract of Salvia blepharophylla
SAB	Sponge associated bacteria
AV	Axinella verrucosa crude extract
AN	pure extract of Angelica
Ace	Small molecule inhibitor
CA	Small molecule inhibitor
JN	Small molecule inhibitor
GS	Small molecule inhibitor
CLO	Small molecule inhibitor

b

Compound	$[M-H]^-$	MS^2	MS^3
6,8-di-C-glucosyl-apigenin	593	503-473-413-353	325
Diglucuronyl-luteolin isomer I	637	351-285	
Glucoronyl-apigenin	621	351-285	
Genipin	225	207-181-165	
Diglucuronyl-luteolin isomer II	637	593-549	505-411
Rosmanol/epirosmanol derivative	389	359-315	
Apigenin derivative	591	560-503-383-268	
Luteolin	285	241-217-199-175-151	147-133-119
Apigenin	269	255-249	
Betulinic acid	455	407	

Supplementary Table S1. (**a**) Codes and description of hits utilized in the platform for the *in vitro* identification of anti-senescent compounds. (**b**) The identified constituents of SH are summarized in table and are mainly apigenin and luteolin glycosides.
